# Supplementary material for: Modeling toes contributes to realistic stance knee mechanics in three-dimensional predictive simulations of walking
Source: PLoS One. 2022 Jan 25;17(1):e0256311. doi: 10.1371/journal.pone.0256311 (PMC8789163; doi:10.1371/journal.pone.0256311)
Supplement: S1 Appendix — (DOCX) [file pone.0256311.s001.docx]

Supplements for

Modeling toes contributes to realistic stance knee mechanics in three-dimensional predictive simulations of walking

Antoine Falisse^1,2*^, Maarten Afschrift^3,4^, Friedl De Groote^2^

^1^ Department of Bioengineering, Stanford University, Stanford, California, USA

^2^ Department of Movement Sciences, KU Leuven, Leuven, Belgium

^3^ Department of Mechanical Engineering, Robotics Core Lab of Flanders Make, KU Leuven, Leuven, Belgium

^4^ Department of Human Movement Sciences, Vrije Universiteit Amsterdam, Amsterdam, Netherlands

* Corresponding author

E-mail: afalisse@stanford.edu (AF)

**Sensitivity analysis about the effect of the toe joint damping value on the simulations.**

We performed a sensitivity analysis to select the damping value of the toe joint. We tested five different values: 0.5, 1, 2, 4.23 (value for a critically damped system given the segment mass and stiffness), and 8.46 (twice the value for a critically damped system). The effect on knee and ankle kinematics and kinetics, plantarflexor activations, and ground reaction forces is depicted in the figure below. Furthermore, the root mean squared errors (RMSEs) with respect to experimental data as well as the effect on the metabolic cost of transport (COT) and the number of iterations for convergence are reported in the table below.

Based on those results, we selected a value of 2 Nm s rad^-1^, which slightly increases RMSE of knee kinematics as compared to lower damping values, but decreases RMSE of knee kinetics, ankle kinematics, and ankle kinetics. When using a value of 2 Nm s rad^-1^, as compared to lower damping values, the first peak of the vertical ground reaction force is overestimated slightly more, but its shape is more similar to experimental data (no double bump as can be observed when zooming in).


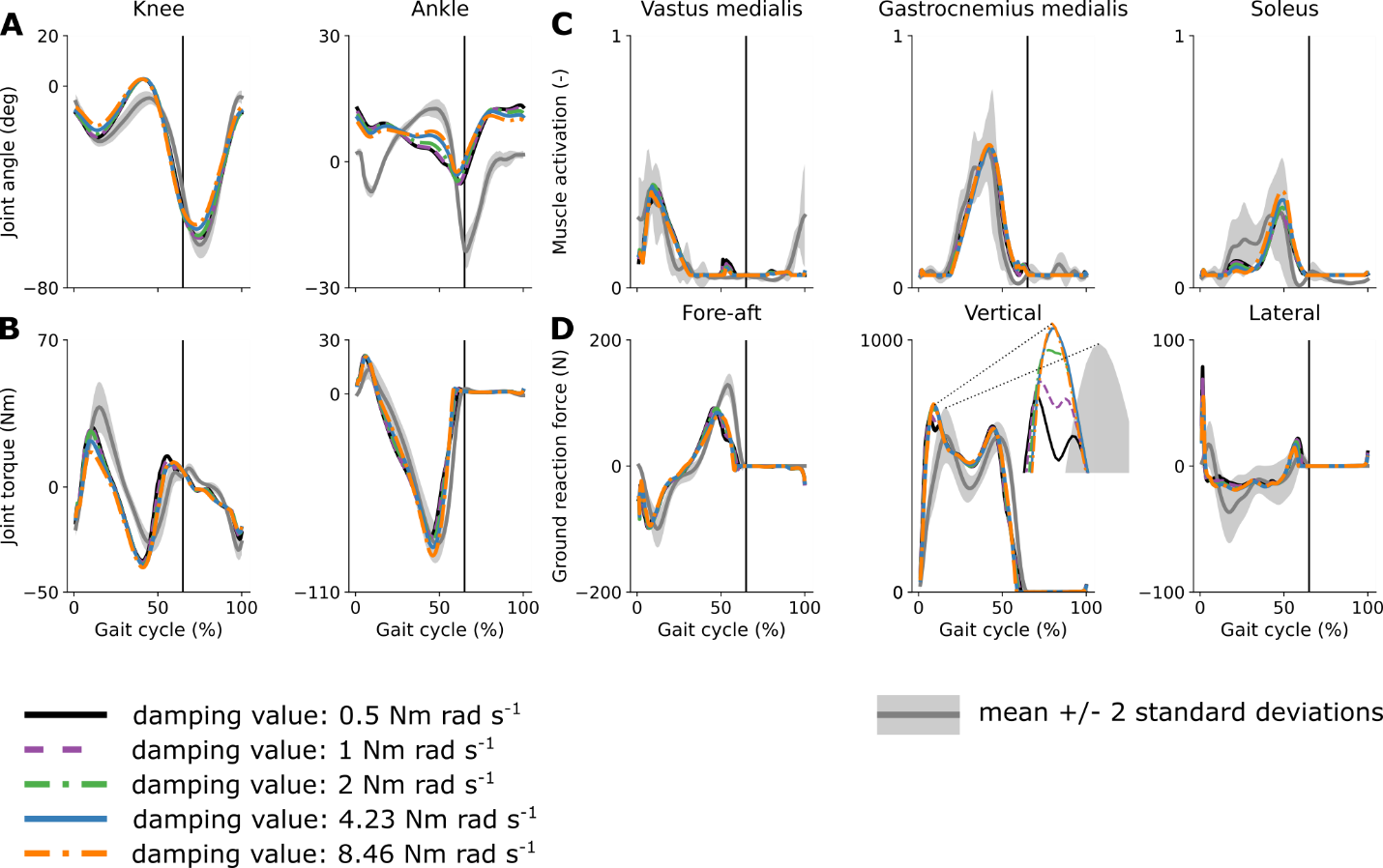


**Figure:** Predicted knee and ankle kinematics (**A**) and kinetics (**B**), muscle activations (**C**), and ground reaction forces (**D**) with the models with different damping values. A zoom is also provided for the first peak of the vertical ground reaction forces to highlight model differences. Experimental data (shaded areas) are shown as mean ± 2 standard deviations. The vertical black lines indicate experimental stance to swing transition.

**Table:** Effect of the damping value on knee and ankle kinematics and kinetics, number of iterations, and metabolic cost of transport (COT). For the RMSEs, the difference with respect to the next lower value expressed as a percentage of the signal’s range is given into squared brackets.

|  |  | Damping value (Nm s rad^-1^) | | | | |
| --- | --- | --- | --- | --- | --- | --- |
|  |  | **0.5** | **1** | **2** | **4.23** | **8.46** |
| Knee | Kinematic  RMSE (deg and  [%difference]) | 4.6 | 4.78 [+0.5%] | 5.26  [+1.4%] | 5.74  [+1.4%] | 6.3  [+1.7%] |
|  | Kinetic  RMSE (Nm and  [%difference]) | 11.52 | 11.15  [-0.6%] | 10.6  [-0.9%] | 11.36  [+1.2%] | 13.11  [+2.7%] |
| Ankle | Kinematic  RMSE (deg and  [%difference]) | 9.07 | 8.74  [-1.1%] | 8.23  [-1.6%] | 7.69  [-1.7%] | 7.35  [-1.1%] |
|  | Kinetic  RMSE (Nm and  [%difference]) | 8.59 | 7.43  [-1.2%] | 6.18  [-1.3%] | 6.79  [+0.6%] | 8.29  [+1.5%] |
| # iterations |  | 1359 | 1207 | 1248 | 1191 | 1202 |
| COT  (J kg^-1^ m^-1^) |  | 4.17 | 4.19 | 4.22 | 4.22 | 4.17 |

**Smooth approximation of the metabolic energy model described by Bhargava et al. [1]**

We implemented a smooth (i.e., twice continuously differentiable) approximation of the metabolic energy model described by Bhargava et al. [1]. Such smooth model is better suited for gradient-based optimization. Note that our implementation originates from the non-smooth OpenSim implementation, which slightly differs from Bhargava’s original implementation. When possible, we indicated where both implementations differ.

In our smooth implementation, we approximated conditional if statements using hyperbolic tangent functions ($\tanh$). For example, the following conditional if statement:

$$y=\left\{ \begin{aligned} a, if x\leq d \\ b, if x>d \end{aligned} \right.$$

can be approximated by:

$$f=0.5+0.5 \tanh\left( b\left( x-d \right) \right),$$

$$y=a+\left( -a+b \right) f,$$

where $b$ is a parameter that determines the smoothness of the transition.

The metabolic energy model includes components for activation heat rate, maintenance heat rate, shortening heat rate, and mechanical work rate. The different components are described below.

**Activation heat rate**

Activation heat rate $\dot{A}$ is described as:

$$\dot{A}\left( t \right)= m \left( \dot{A}_{fast}u_{fast}\left( t \right)+\dot{A}_{slow}u_{slow}\left( t \right) \right),$$

where $=1$ (Bhargava et al. used a decay function, whereas we used a constant based on [2] as in OpenSim), $m$is the muscle mass, $\dot{A}_{fast}=133$ and $\dot{A}_{slow}=40$ are the activation heat rate constants for fast and slow twitch fibers, and $u_{fast}$ and $u_{slow}$ are the excitation levels of the fast and slow twitch fibers:

$$u_{fast}\left( t \right)=r_{fast}\left( 1-\cos\left( \frac{}{2}e\left( t \right) \right) \right),$$

$$u_{slow}\left( t \right)=r_{slow}\left( \sin\left( \frac{}{2}e\left( t \right) \right) \right),$$

where $r_{fast}=1-r_{slow}$ and $r_{slow}$ are the ratios of fast and slow twitch fibers obtained from [3], and $e$ is muscle excitation. Note that in practice, we use muscle activation instead of muscle excitation in those equations, since we use an implicit formulation of muscle activation dynamics and hence do not have access to muscle excitation directly (muscle excitation can be computed post-processing).

**Maintenance heat rate**

Maintenance heat rate $\dot{M}$ is described as:

$$\dot{M}\left( t \right)=L\left( t \right) m \left( \dot{M}_{fast}u_{fast}\left( t \right)+\dot{M}_{slow}u_{slow}\left( t \right) \right),$$

where $L=l_{m}$ (note that Bhargava et al. used a function that models the dependence on fiber length $l_{m}$, whereas we use fiber length directly to avoid the use of a highly discontinuous function [4]), and $\dot{M}_{fast}=111$ and $\dot{M}_{slow}=74$ are the maintenance heat rate constants for fast and slow twitch fibers.

**Shortening heat rate**

The shortening heat rate model differs between concentric and eccentric contractions. We smoothed the transition between both contraction types using our smoothing function. Shortening heat rate $\dot{S}$ is described as:

$$\dot{S}\left( t \right)=-\alpha\left( t \right) v_{m}\left( t \right),$$

where $v_{m}$ is fiber velocity ($v_{m}>0$ indicates muscle lengthening) and $\alpha$ is given by:

$$\alpha\left( t \right)=\beta\left( t \right)+\left( -\beta\left( t \right)+0.157 f_{m}\left( t \right) \right) v_{m}^{+}\left( t \right),$$

where total fiber force $f_{m}$ is the sum of active and passive fiber force, and:

$$\beta\left( t \right)=0.16 f_{iso}\left( t \right)+0.18 f_{m}\left( t \right),$$

$$v_{m}^{+}\left( t \right)=0.5+0.5 \tanh\left( b_{s} v_{m}\left( t \right) \right),$$

where $b_{s}=10$ is a smoothing constant ($v_{m}^{+}$ is close to zero when the muscle is shortening: $v_{m}<0$) and $f_{iso}$ is the fiber force that would be developed at the current activation level and fiber length under isometric conditions:

$$f_{iso}\left( t \right)=a\left( t \right) f_{a, l}\left( t \right),$$

where a is muscle activation and $f_{a, l}$ is the fiber force from the active force-length relationship.

**Mechanical work rate**

We excluded negative mechanical work (i.e., work rate resulting from eccentric contraction) in our smooth implementation. To this aim, we smoothed the transition between positive rate and zero using our smoothing function. Mechanical work rate $\dot{W}$ is therefore described as:

$$\dot{W}\left( t \right)=-\left( f_{a}\left( t \right) v_{m}\left( t \right) v_{m}^{-}\left( t \right) \right),$$

where $v_{m}^{-}=1-v_{m}^{+}$ ($v_{m}^{-}$ is close zero when the muscle is lengthening: $v_{m}>0$), such as to exclude negative mechanical work.

**Total heat rate**

Following the OpenSim implementation, we included a clamping that prevents the total metabolic rate to be negative. This clamping is done by increasing the shortening heat rate. We smoothed the transition between positive and negative total metabolic rate using our smoothing function:

$$\dot{E}_{c}\left( t \right)=\dot{A}\left( t \right)+\dot{M}\left( t \right)+\dot{S}\left( t \right)+\dot{W}\left( t \right),$$

$$\dot{E}_{c}^{-}\left( t \right)=0.5+0.5 \tanh\left( {-b}_{c}\dot{E}_{c}\left( t \right) \right),$$

$$\dot{S}_{c}\left( t \right)=\dot{S}\left( t \right)-\left( {\dot{E}_{c}\left( t \right) \dot{E}}_{c}^{-}\left( t \right) \right),$$

where $b_{c}=10$ is a smoothing constant ($\dot{E}_{c}^{-}$ is close to zero when $\dot{E}_{c}>0$).

We included another clamping that prevents the total heat rate for a given muscle to fall below 1 W kg^-1^ [5]. We smoothed the transition between total heat rate higher and lower than 1 W kg^-1^ using our smoothing function. The total heat rate $\dot{H}_{m}$ (expressed in W kg^-1^) is given by:

$$\dot{H}_{m}\left( t \right)=\frac{\dot{A}\left( t \right)+\dot{M}\left( t \right)+\dot{S}_{c}\left( t \right)}{m},$$

and the clamping formulation is given by:

$$\dot{H}_{m,c}\left( t \right)=\dot{H}_{m}\left( t \right)+\left( -\dot{H}_{m}\left( t \right)+1 \right)\left( 0.5+0.5 \tanh\left( b_{c}\left( 1-\dot{H}_{m}\left( t \right) \right) \right) \right),$$

$$\dot{H}_{c}\left( t \right)=m \dot{H}_{m,c}\left( t \right),$$

where $\dot{H}_{c}$ is the clamped total heat rate expressed in W.

**Metabolic energy rate**

The metabolic energy rate is finally given by:

$$\dot{E}\left( t \right)=\dot{H}_{c}\left( t \right)+\dot{W}\left( t \right),$$

where $\dot{E}$ is the term minimized in the objective function (see equation (1) in main manuscript).

**References**

1. Bhargava LJ, Pandy MG, Anderson FC. A phenomenological model for estimating metabolic energy consumption in muscle contraction. J Biomech. 2004;37(1):81–8.

2. Anderson FC, Pandy MG. A dynamic optimization solution for vertical jumping in three dimensions. Comput Methods Biomech Biomed Engin. 1999;2(764699363):201–31.

3. Uchida TK, Hicks JL, Dembia CL, Delp SL. Stretching your energetic budget: how tendon compliance affects the metabolic cost of running. PLoS One. 2016;11(3):e0150378.

4. Falisse A, Serrancolí G, Dembia CL, Gillis J, Jonkers I, De Groote F. Rapid predictive simulations with complex musculoskeletal models suggest that diverse healthy and pathological human gaits can emerge from similar control strategies. J R Soc Interface. 2019;16(157):20190402.

5. Umberger BR, Gerritsen KGM, Martin PE. A model of human muscle energy expenditure. Comput Methods Biomech Biomed Engin. 2003;6(2):99–111.
