# Supplementary material for: Modeling toes contributes to realistic stance knee mechanics in three-dimensional predictive simulations of walking
Source: PLoS One. 2022 Jan 25;17(1):e0256311. doi: 10.1371/journal.pone.0256311 (PMC8789163; doi:10.1371/journal.pone.0256311)
Supplement: S1 Table — (DOCX) [file pone.0256311.s006.docx]

**S1 Table: Influence of the mesh density on the convergence profile.**

|  | | N=50 | | N=75 | | N=100 | | N=125 | |
| --- | --- | --- | --- | --- | --- | --- | --- | --- | --- |
|  |  | # Iter | Cost | # Iter | Cost | # Iter | Cost | # Iter | Cost |
| With  toe joints | Hot-start | 776 | 355.6 | 971 | 352.2 | 1248 | 350.1 | 1978 | 350.5 |
|  | Cold-start | 815 | 352.8 | 1051 | 351.0 | 849 | 351.3 | 991 | 350.0* |
| Without toe joints | Hot-start | 917 | 290.7 | 969 | 288.4 | 1860 | 287.3 | 2097 | 286.7 |
|  | Cold-start | 1012 | **344.6** | 1197 | 288.3 | 894 | 287.5 | 988 | 287.0* |

N is the number of mesh intervals, # Iter is the number of iterations before convergence, and Cost is the optimal cost value (integral of equation 1 with the optimal values). Bold values indicate large differences between both initial guesses and * indicates solutions with the lowest optimal cost values.
