# Supplementary material for: Modeling toes contributes to realistic stance knee mechanics in three-dimensional predictive simulations of walking
Source: PLoS One. 2022 Jan 25;17(1):e0256311. doi: 10.1371/journal.pone.0256311 (PMC8789163; doi:10.1371/journal.pone.0256311)
Supplement: S2 Table — (DOCX) [file pone.0256311.s007.docx]

**S2 Table: Influence of the convergence tolerance on the convergence profile.**

|  | | Tolerance = 1e^-4^ | | Tolerance = 1e^-5^ | | Tolerance = 1e^-6^ | |
| --- | --- | --- | --- | --- | --- | --- | --- |
|  |  | # Iter | Cost | # Iter | Cost | # Iter | Cost |
| With  toe joints | Hot-start | 1248 | 350.1 | 2045 | 350.1 | 4043** | 350.1 |
|  | Cold-start | 849 | 351.3 | 2499 | 349.9 | 3967** | 349.9* |
| Without toe joints | Hot-start | 1860 | 287.3 | 2894 | 287.2 | 3820 | 287.2* |
|  | Cold-start | 894 | 287.5 | 2782 | 287.2 | 3753 | 287.2 |

Tolerance refers to the IPOPT convergence tolerance, # Iter is the number of iterations before convergence, and Cost is the optimal cost value (integral of equation 1 with the optimal values). * indicates solutions with the lowest optimal cost values. ** indicates solutions that were not optimal and for which the IPOPT restoration phase failed.
