# Supplementary material for: Modeling toes contributes to realistic stance knee mechanics in three-dimensional predictive simulations of walking
Source: PLoS One. 2022 Jan 25;17(1):e0256311. doi: 10.1371/journal.pone.0256311 (PMC8789163; doi:10.1371/journal.pone.0256311)
Supplement: Attachment — Submitted filename: Response To Reviewers.docx [file pone.0256311.s010.docx]

Dear Editor,

Many thanks for giving us a chance to submit a revised version of our manuscript. We have addressed all comments of the reviewers. Some of our results slightly changed when re-running simulations to answer some of the reviewers’ questions. We found that using a higher toe joint damping value produced simulations that better reproduced experimental data. In the revised version of this manuscript, we therefore present slightly updated results, and we adjusted our statements in different parts of the manuscript to reflect our new results. We believe our paper improved by a lot with this round of review and would like to thank the reviewers for all the comments they made. We hope the paper now satisfies the standards of the journal, but we would be happy to further clarify certain parts if deemed necessary. You will find our answers to the reviewers’ questions in blue below, with modified text in red.

Yours sincerely,

Antoine Falisse on behalf of all co-authors.

Reviewer #1: This manuscript describes a development of a musculoskeletal model of human lower body. The authors altered the mass distribution, skeletal and muscular properties of the foot and investigated their influence on the modelled human walk. This is a technical report of good quality, however, there are a several issues that need to be addressed before publication.

We would like to thank the reviewer for the thorough review. We have addressed all comments and answered all questions as detailed below. We would like to mention that when addressing the question of the reviewer regarding how we selected the toe joint damping value, we found that our simulations got better (i.e., better replicated experimental data) when using higher values (see details in the answer to the question), and that our analysis of the effect of the Achilles tendon stiffness slightly changed (we obtained better kinematics when decreasing the Achilles tendon stiffness). We have adjusted our results and statements to reflect those changes as detailed below. We also added supplementary information to document our observations.

Major:

1. The introduction does not provide enough justification of why specifically it is important to investigate the influence of foot complexity and Achilles tendon on walking, with the latter raising even more questions when it does not yield a positive result. The lack of ankle plantarflexion and knee flexion are mentioned, but it is unclear why these features of human gait are important. Is there any evidence that the lack of them leads to instability or them being present is a symptom?

We agree with the reviewer that our introduction lacked justification of why investigating those specific features is relevant to address the lack of stance ankle plantarflexion and knee flexion. We especially agree that our justification regarding looking at the Achilles tendon stiffness was too limited. We have addressed that in the revised version of the manuscript. We believe modeling toes does not need much more justification, since, as mentioned already, there is a *“clear extension of the toe joints during walking”*, which cannot be captured by single-segment foot models, providing, in our opinion, enough of a justification to look at the effect of more complex foot models on predictive simulations.

Whether the observed discrepancies between measured and predicted kinematics are important for human gait stability (and other human gait parameters) should, in our opinion, be left to the judgment of the readers. Our goal with this work is to generate simulations that reproduce as closely as possible human gait. Not being able to reproduce human gait features suggests that something is missing in our predictive models, and we believe it is important to understand what might be missing in order to make our predictive models better over time such that they can be used to answer questions such as “What is the effect of an exoskeleton?”, “What is the effect of a specific treatment?”, or “What is the effect of a specific training program?” We also believe that improving the realism of our simulations is relevant for understanding the fundamentals of gait neuromechanics. As long as our simulations are not closer to measured data, it is hard to answer scientific questions with confidence using predictive models.

We agree with the reviewer that our analysis of the effect of Achilles tendon stiffness requires justification. As detailed below, we now provide more details about why we looked at Achilles tendon stiffness in particular. Furthermore, as mentioned above, when using a higher damping value, we obtained better ankle kinematics when decreasing the Achilles tendon stiffness, making those results even more relevant.

In the revised version of our paper, we made clearer why specifically we looked at the Achilles tendon stiffness. As was already introduced, the Achilles tendon, the longest tendon of the human body, is known to play a major role in energy savings during gait by storing and releasing energy [1–4]. It is therefore crucial to get the tendon model parameters right if we want to predict human gait by optimizing a performance criterion that includes metabolic energy consumption and muscle activations. Both metabolic power and muscle activity are widely believed to depend on Achilles tendon stiffness for the plantarflexors and plantarflexors have a considerable (estimated 30%) contribution to whole body energy consumption during walking. In our musculoskeletal models, tendon stiffness scales with maximal isometric force and inversely with tendon slack length, derived from [5]. Yet these scaling rules might not represent reality. In a previous study, we found that we had to make the Achilles tendon more compliant (by decreasing the stiffness value by about 60%) to be able to track gastrocnemius fiber lengths measured using ultrasonography during walking [6], suggesting that the default value might not be correct. In another of our previous studies, we also had to make the Achilles tendon more compliant to achieve realistic inverse dynamic estimates of gastrocnemius fiber length for human running [7]. Another motivation for looking at Achilles tendon stiffness is that the gastrocnemii, which are attached to the Achilles tendon, cross both ankle and knee joints, which are the joints for which we do not predict the kinematics well with our models. We hoped that by better modeling the Achilles tendon, we could improve the predicted kinematics of those joints.

Changes in revised introduction (lines: 51-54):

*Understanding why predictive simulations do not capture those gait features well will advance our knowledge of gait neuromechanics and is key to improving the realism of predictive simulations, which in turn is important to enable the use of simulations for designing gait interventions in clinical settings.*

Changes in revised introduction (lines: 95-109):

*First, Achilles tendon compliance has been suggested to improve gait efficiency both by allowing for storage and release of energy throughout the gait cycle and through its influence on the operating length and velocity of the plantarflexors* [1–4,8]*. Since it is the interaction between ankle kinematics and Achilles tendon properties that determines plantarflexor operating length and velocity, and thereby efficiency, adjusting the Achilles tendon stiffness in our simulations that optimize efficiency might influence predicted ankle kinematics. Second, we previously found that reducing Achilles tendon stiffness in our models resulted in inverse dynamic estimates of gastrocnemius fiber length trajectories that were in closer agreement with fiber length trajectories measured using ultrasonography during walking* [6] *and running* [7]*. In inverse dynamic simulations of walking, we obtained the best agreement between simulated and measured fiber lengths when decreasing Achilles tendon stiffness by 60%. Finally, since the gastrocnemii, attached to the Achilles tendon, cross both knee and ankle joints, we expected changes in Achilles tendon stiffness to affect both joints and therefore to potentially improve our kinematic predictions at both levels. We found that decreasing the Achilles tendon stiffness by up to 60% slightly improved predictions of ankle kinematics.*

2. Many of the statements about differences between models are made based on qualitative evaluation of the figures, without quantification. In most cases, it is not very visible on plots with 5 lines being very close to each other, and can be supported simply with a correlation, which will provide an exact value for the lack of difference or presence of small differences.

We agree with the reviewer that our analysis lacked quantitative evaluations. In the revision, we used root mean squared errors (RMSE) between our predictions and mean experimental data to quantify kinematic and kinetic changes between different models. We also reported the difference in RMSE expressed as a percentage of the signal’s range. We used RMSE instead of correlations as we found them more meaningful in this context given that they are expressed in the signal’s unit.

Changes in revised methods (lines: 244-247):

*For all results, we quantified the effect of the changes using root mean squared error (RMSE) of stance knee and ankle kinematics and kinetics with respect to mean experimental data. Furthermore, we quantified changes between models by expressing the difference in RMSE as a percentage of the signal’s range during stance.*

Changes in revised results (lines: 252-256):

*Adjusting the model mass distribution improved stance knee kinematics and kinetics (RMSE decreased from 10.2 to 8.6° [-4.6% when expressed as a percentage of the signal’s range] and from 22.1 to 20.3 Nm [-2.7%], respectively), had barely any effect on ankle kinematics (no change in RMSE) and kinetics (RMSE increased from 15.0 to 15.4 Nm [0.4%]), and had a small effect on muscle activations (Fig 1).*

Changes in revised results (lines: 258-262):

*Moving the contact spheres higher up further improved stance knee kinematics and kinetics (RMSE decreased from 8.6 to 6.8° [-5.5%] and from 20.3 to 17.8 Nm [-3.8%], respectively) by producing higher knee flexion angles, knee extension torques, and knee extensor activations during stance. Ankle kinematics and kinetics slightly worsened with higher contact spheres (RMSE increased from 7.3 to 7.5° [0.8%] and from 15.4 to 17.0 [1.7%]).*

Changes in revised results (lines: 282-291):

*Predictive simulations with a model incorporating toe joints produced stance knee flexion angles, knee extension torques, and ankle flexion torques that better reproduced experimental data as compared to simulations produced with a model with locked toe joints (RMSE decreased from 6.8 to 5.3° [-4.5%], from 17.8 to 10.6 Nm [-11.2%], and from 17.0 to 6.2 Nm [-11.2%] for the stance knee kinematics, knee kinetics, and ankle kinetics, respectively) (Figs 1-2; results for all joints and muscles are shown in S1-3 Figs; animations are shown in S1-2 Movies). The predicted stance ankle kinematics changed when incorporating toe joints but slightly worsened compared to experimental data (RMSE increased from 7.5 to 8.2° [2.4%]). Ground reaction forces produced with models with and without toe joints were qualitatively comparable, although incorporating toe joints decreased the first peak of the vertical forces (from 761 to 725 N), increasing its agreement with experimental data (620 N).*

3. The Results related to optimization evaluation, e.g., mesh density and tolerance, are very important for model validation and reproduction, but are not generalizable, as authors specify themselves, and would be more appropriate for Methods.

We agree with the reviewer that the analysis of convergence is better suited for the methods, and we moved it there in the revised version of the manuscript. Yet we kept the interpretation of this analysis in the discussion, as we believe it is relevant to highlight the importance of doing such analysis when using trajectory optimization in biomechanics. With the rise of user-friendly tools for trajectory optimization in biomechanics, such as OpenSim Moco [9], users might be unaware of underlying modeling and formulation choices (initial guess, convergence tolerance, number of mesh intervals, etc.) and we believe it is important to highlight such choices and their implications in the discussion, as they might be overlooked if kept only in the methods. Please note that we moved the two tables associated with the convergence analysis to the supplements.

Changes in methods (lines: 190-204)

*We performed a convergence analysis by evaluating the sensitivity of the results to the number of mesh intervals (50, 75, 100, and 125 mesh intervals per half gait cycle) and to IPOPT convergence tolerance (10^-4^ [default], 10^-5^, and 10^-6^). Increasing the mesh density had barely any influence on the results (i.e., state and control trajectories) (S1 Table), suggesting a good enough simulation accuracy. Yet it is worth noting that both initial guesses led to different solutions when using only 50 mesh intervals for models without toes, highlighting the need for different initial guesses. With higher mesh densities, both initial guesses led to very similar solutions. Overall, the number of iterations increased when increasing the mesh density, but with no clear pattern. At high mesh densities (100 and 125), the number of iterations was lower for the cold-start than for the hot-start. Tightening the convergence tolerance also had barely any influence on state and control trajectories (S2 Table), suggesting that the default tolerance of 10^-4^ is strict enough. On average, the number of iterations was more than twice and thrice as large for a convergence tolerance of 10^-5^ vs 10^-4^ and 10^-6^ vs 10^-4^, respectively. For all results of this study, we used 100 mesh intervals per half gait cycle (the dynamic equations were therefore satisfied at 300 collocation points) and a convergence tolerance of 10^-4^.*

Minor:

L47-49: This needs a citation or a description of the effect.

We added a citation to our previous work that discusses this statement. Please note that this challenge related to predicting knee flexion during stance is also described in length later in the introduction, with references to many other papers.

L50: Could you describe what is the terminal stance exactly? Including a description of the major states within step cycle might help a novice reader navigate.

We agree that using this terminology was confusing, especially since ankle kinematics is badly predicted during the entire stance phase. We therefore removed references to terminal stance in the revised version of the manuscript, and only talk about stance, which we believe will improve the clarity of the paper. Since we now only talk about stance and swing, we believe there is no need for further clarification.

L71-74: A very long sentence, consider splitting.

Thank you for the suggestion. This was done in the revised version.

L117: How much of a change is it from the generic model? Is there anatomical evidence to support either of these orientations?

In their paper [10], Delp et al. reported that they modeled the metatarsophalangeal joint based on [11], which used as axis the line connecting the heads of the second and fifth metatarsal bones (angle of 62°, range 53°-72°, with respect to the axis of the foot). Yet Delp et al. reported unrealistic motion of the metatarsophalangeal joint with the phalanges separating from the metatarsals. They therefore rotated the joint (-8°) about the vertical axis to minimize disarticulation. We agree that the joint definition from Delp et al. minimized disarticulation. Yet empirical observation (looking at feet during walking) showed that the toes do not rotate about a common axis through the base of the metatarsals when walking. Functionally, the toes seem to bend around an axis perpendicular to the sagittal plane. Therefore, we assumed the toes to rotate about an axis perpendicular to the midfoot at the base of the big toe. Note that this is a very simplistic representation of the foot, which in reality is made of 30 joints (including five toe joints). Our goal in this study was to show the impact of including versus locking the metatarsophalangeal joint rather than to accurately model it. The latter goal would need a more expressive food model, and developing such models for predictive simulations was out of the scope of this study. We have revised this part of the methods to better motivate our change.

Changes in the methods (lines: 127-132)

*We empirically found that an orientation of the toe joint axis perpendicular to the sagittal plane in the anatomical position better reproduced the movement of the toes during walking as compared to the generic orientation of the toe joint axis through the base of the metatarsals. We confirmed through preliminary simulations that using a toe joint axis that was perpendicular to the sagittal plane resulted in simulated walking patterns that were in better agreement with experimental data than using the generic toe joint axis.*

L122: How were these specific values chosen?

The stiffness value was based on literature [12].

Regarding the damping value, we performed a sensitivity analysis for the review of this paper. We found that our simulations better reproduced experimental data with a higher damping value than the one we originally used. The results of the sensitivity analysis are detailed in S1 appendix. In the revision of this paper, we therefore used a higher damping value (2 instead of 0.5 Nm s rad^-1^). Using a higher damping value had a clear effect on simulations with lower Achilles tendon stiffness, highlighting how mechanical parameters are linked to each other. We have adjusted the parts of the paper related to results of the analysis of the effect of the Achilles tendon stiffness.

Changes in the abstract (lines: 30-32)

*Decreasing Achilles tendon stiffness improved the realism of ankle kinematics, but there remain large discrepancies with experimental data.*

Changes in the introduction (lines: 108-109)

We found that decreasing the Achilles tendon stiffness by up to 60% slightly improved predictions of ankle kinematics.

Changes in the methods (lines: 135)

*We selected the damping value from a sensitivity analysis (S1 Appendix).*

Changes in the results (lines: 314-319)

*Decreasing Achilles tendon stiffness by up to 60% improved stance ankle kinematics (Fig 3), with largest improvements with a 60% decrease (RMSE decreased from 8.2 to 6.3 Nm [-6.1%]. Decreasing Achilles tendon stiffness by 60% barely affected knee kinematics, but slightly worsened knee and ankle kinetics (RMSE increased from 10.6 to 13.2 [4%] and from 6.2 to 8.1 [2%], respectively). Decreasing the Achilles tendon stiffness by more than 60% had a large effect on the knee and ankle kinematics and kinetics during stance. Soleus activity increased and turned on earlier with decreasing tendon stiffness.*

Changes in the discussion (lines: 342-348)

*our study reveals that a more physiologically plausible model of the feet, which incorporate toe joints, contributes to robustly eliciting knee flexion during stance but also improves stance ankle kinetics and reduces overestimation of the first vertical ground reaction force peak. Adding toe joints slightly worsened predictions of ankle kinematics, which were already of limited accuracy when using a model without toe joints. Decreasing Achilles tendon stiffness by up to 60% slightly improved the realism of the simulated ankle kinematics, but further reduction drastically altered the simulated gait pattern.*

Changes in the discussion (lines: 417-422)

*We also found that passive damping in the toe joints interacted with Achilles tendon stiffness. When damping was reduced, smaller decreases in Achilles tendon stiffness (more than 20%) elicited large changes in kinematics (S4 Fig). Yet reducing damping had little effect on fascicle lengths. Even though reducing Achilles tendon stiffness improved stance ankle kinematics, differences with experimental data remained large, in particular at the beginning and end of the stance phase.*

Changes in the discussion (lines: 431-435)

*We obtained the best fits between simulated and measured fiber lengths when reducing Achilles tendon stiffness by on average 60%, which is in agreement with the results from the predictive simulations of the present study. Note that fiber length tracking errors remained relatively high in those inverse simulations with reduced Achilles tendon stiffness.*

Changes in the conclusion (lines: 463-469)

*Incorporating toes in the musculoskeletal model contributed to robustly eliciting stance knee flexion, improved ankle and knee kinetics, and normalized the first vertical ground reaction force peak. Yet it did not improve stance ankle kinematics. In contrast, decreasing the stiffness of the Achilles tendon by up to 60% slightly improved stance ankle kinematics, but there remain large discrepancies with experimental data. The poor predictions of ankle kinematics in simulations of walking thereby remain an open question, and further work is required to better understand how mechanical factors shape human walking.*

L138: What technology was used to record 3D marker positions, which devices were used for EMG recordings, how were ground reaction forces recorded?

We have revised our manuscript to answer those questions.

Changes in methods (lines: 152-156)

*Marker coordinates were recorded (100 Hz) using a ten-camera motion capture system (Vicon, Oxford, UK), ground reactions forces were recorded (1000 Hz) using two force plates (AMTI, Watertown, USA), and electromyography was recorded (1000 Hz) using a wireless acquisition system (ZeroWire EMG Aurion, Milano, Italy) from 24 lower limb muscles.*

L145: How many and which muscles were recorded?

We recorded EMGs from 24 muscles using an 16-channel system and an 8-channel system: lateral and medial hamstrings (left and right), tibialis anterior (left and right), peroneus brevis (left) and longus (left and right), gastrocnemius medialis (left) and lateralis (left and right), soleus (left and right), vastus lateralis and medialis (left and right), adductor longus (left), rectus femoris (left and right), TFL (left), and gluteus medius (left and right).

We specified the number of muscles in the revised version of the methods. We think listing all 24 muscles densify the text a lot while not adding much to the story. We therefore only indicated that those were 24 muscles in the lower limbs. Please note that electromyography was only used to evaluate the simulated muscle activations as now explicitly mentioned.

Changes in methods (lines: 154-156)

*...and electromyography was recorded (1000 Hz) using a wireless acquisition system (ZeroWire EMG Aurion, Milano, Italy) from 24 lower limb muscles.*

Changes in methods (lines: 163)

…and used for evaluating simulated muscle activations.

L145: The EMG was first filtered on two non-overlapping bands: first 20-400 Hz, and then <10Hz. There should be minimal signal left. Are there examples of the prior use of this method?

We rectified the signal in between applying the two filters. This approach is standard in the field (see for example [13] or [14]) The first filter is applied to remove movement noise and the second one is applied to get an idea of the signal amplitude.

L160: This section should provide a specific formula for the metabolic energy rate, as well as the quality of approximation.

The formula for the metabolic energy rate is described in length in the cited publication [15], and we believe it is out of the scope of this paper to provide it again here, as it involves many terms with many variables that would need to be defined. Yet we revised this part of the methods to make clearer what terms were included in the computation of the metabolic energy rate.

Changes in methods (lines: 176-179)

*We modeled metabolic energy rate* $\dot{E}$ *using a smooth approximation of the phenomenological model described by Bhargava et al.* [15]*, which describes metabolic energy rate as the sum of five terms: muscle activation, shortening, and maintenance heat rates, mechanical work rate, and basal metabolic rate.*

L180: Why was the generic torso mass changed?

In our previous study and publication [16], we had started working with a model without arms (generic gait2392 model from OpenSim). We then added arms to the model, using the arms from the model of Hamner et al. [17]. However, when adding the arms, we did not change the mass of the torso (nor the mass of the other segments), whereas Hamner et al., in their model, distributed part of the mass of the torso to the arms, resulting in a model with the same overall mass as the generic gait2392 model but with a lighter torso relative to the lower limbs. In the present study, we started from the generic Hamner model, which hence had a lighter torso as compared to the model of our previous study. We believe both generic (and thus scaled) models are representative for a subgroup of healthy adults, as the mass of the torso is very dependent on the individual (e.g., in an extreme case, obese people tend to have a heavier torso as compared to their lower limbs, but that would not be reflected in a scaled OpenSim model for which the mass distribution of the generic model is preserved when scaling; the scaled model of an obese individual would thus not have a heavier torso as compared to the lower limbs).

Changes in methods (lines: 209-2014)

*In our previous study, we extended OpenSim’s gait2392 model with arms without adjusting the mass of all other segments resulting in a generic (i.e., pre-scaling) model with a torso of 34.2 kg. Here, we started from the generic model proposed by Hamner et al.* [17]*, which is also based on OpenSim’s gait2392 model and is more commonly used. In Hamner’s model the mass of the trunk was reduced when adding arms, resulting in a generic model with a torso of 26.8 kg.*

L220-228: These statements should be supported by quantified measurements that specify how much the model results differ, e.g., a correlation between traces, possibly in Methods.

We agree. As mentioned in the response above, changes were quantified in the revised version of the paper.

Figures 1 and 2 are in the wrong order.

Thanks for noticing that. It looks like we uploaded the figures in the wrong order. We will make sure they are uploaded properly upon re-submission.

Figure 2A legend would benefit from specifying shaded region as experimental data.

We have added the experimental data as part of the legends of all relevant figures.

Table 1. Would benefit from including the experimental metabolic cost for comparison.

We have added a column with reference data following the suggestion of the reviewer.

L246-252: Same for quantification of changes.

We agree. As mentioned in the response above, changes were quantified in the revised version of the paper.

The result that the vertical GRF has smaller peak in models with toes should be highlighted more as a benefit of toe model, although leading to higher metabolic cost. It seems to be important enough to be highlighted in the discussion, conclusion and abstract.

We agree with the reviewer and thank them for their suggestion. We have highlighted this result in the revised abstract, results, and discussion sections. Please note that when using a higher damping value, the first vertical ground reaction force peak was higher than with the original damping value. Yet we previously observed a double bump at the first peak, which disappeared when using a higher damping value, making the shape of the simulated curve more like its experimental counterpart (see figure in S1 Appendix).

Changes in abstract (lines: 28-29)

*Modeling toes also slightly decreased the first vertical ground reaction force peak, increasing its agreement with experimental data*

Changes in results (lines: 290-291)

*although incorporating toe joints decreased the first peak of the vertical forces (from 761 to 725 N), increasing its agreement with experimental data (620 N)*

Changes in discussion (lines: 344-345)

*and reduces overestimation of the first vertical ground reaction force peak*

Changes in conclusion (lines: 464-465)

*and normalized the first vertical ground reaction force peak*

What was the main contributor to the increased metabolic cost?

The quadriceps (vasti and rectus femoris) contributed for ~29% to the increase in metabolic cost. We added this quantitative result to the revised results. There was already a section in the discussion about this: *“The increased metabolic energy expenditure in the knee extensors is in line with previous observations that knee flexion during stance is energetically costly [4]”.*

Changes in results (lines: 295-296)

*The quadriceps (vasti and rectus femoris) mainly contributed to the increase in metabolic cost of transport (29% of the increase).*

L272-282: It would be interesting to see which values of the generic tendon stiffness are closer to the physiological behavior curves.

We agree with the reviewer that this would be very valuable to see/know. Based on our previous study, a normalized value of 14 seems closer to physiology (default is 35), since it allowed better tracking of experimental gastrocnemius fiber lengths measured using ultrasonography [6]. Interestingly, in our new results (with a higher damping value) this is also the value we found to best predict ankle kinematics. We have made that clearer in the revised version of the manuscript.

Changes in discussion (lines: 431-435)

*We obtained the best fits between simulated and measured fiber lengths when reducing Achilles tendon stiffness by on average 60%, which is in agreement with the results from the predictive simulations of the present study.*

What is the scientific purpose of mesh density and tolerance measurements? Can this be moved to Methods?

We agree with the reviewer as mentioned above already. We moved that part to the methods.

L357: Are there any comparative studies supporting this?

We do not have comparative studies to back up this statement, but this is our expectation based on observing (a lot!) people walking. Walking with straight knees at a normal/preferred walking speed is very challenging, and we do not expect people to change their walking pattern so much when walking with thicker soles. We made sure we wrote in the manuscript that this was an expectation.

L371: Stability can also be produced by coactivation of antagonistic muscles, which act in anticipation of a perturbation. This should be mentioned, e.g. with references to Hogan 1984 in IEEE, Stroeve 1999 in Bio Cybernetics.

Thanks for the pointer to those studies. We now mention coactivation of antagonist muscles as part of our discussion on stability and cite the two papers you suggested.

Changes in discussion (lines: 382-383)

*Stability can be obtained through increased impedance by coactivation of antagonist muscles* [18,19] *and/or feedback control to react to unexpected perturbations*

Source code contains models for two subjects: <https://github.com/antoinefalisse/predictsim_mtp/tree/master/OpenSimModel>. Could you specify which one was used?

Thanks for looking at the source code. Since submission, we deeply cleaned the repository. You can now find folders with the new and old models, corresponding to the terminology used in the paper, in the folder OpenSimModel. We have tried to document the source code and repository as well as possible so that others can reproduce our results and build on top of our work. Note that the cleaned repository is at the moment in the *cleaning* branch on Github.

Reviewer #2: This work is slightly modified published simulation work by the same authors. Authors added the toes and metatarsophalangeal joints. Authors claim that this modification “contributed to robustly eliciting physiological stance knee flexion angles, knee extension torques, and knee extensor activity.”

The reviewer is right that this work builds on our previous work. Our previous study left us wondering about how we could further improve our predictions of stance knee flexion and ankle kinematics. Here we present the key investigations we performed with the aim of improving knee and ankle kinematics.

We would like to point out that when performing a sensitivity analysis to answer a question of another reviewer, we found that using a higher toe joint damping value produced better knee and ankle kinetics, and ankle kinematics than when using the original value. Using this higher damping value when studying the effect of the Achilles tendon stiffness also had a big impact, as our predictions of ankle kinematics actually improved with a more compliant tendon. In the revised version of our manuscript, we have therefore adjusted our results and statements to reflect those changes. We thank the reviewer for the comments they provided.

However, at the same time ankle joint angle became worse at the middle of gait (see S1Fig). It is not clear why reproducing better knee joint angle is more important than ankle joint angle.

We agree with the reviewer that stance ankle kinematics became worse when adding toes. Yet when using a higher damping value (as mentioned above), this negative effect was quite minor (RMSE increased from 7.5 to 8.2°, which corresponds to only 2.4% of the signal’s range) given the benefits for the knee kinematics and kinetics, and for the ankle kinetics (RMSE decreased from 6.8 to 5.3° [-4.5%], from 17.8 to 10.6 Nm [-11.2%], and from 17.0 to 6.2 Nm [-11.2%] for the stance knee kinematics, knee kinetics, and ankle kinetics, respectively - the value between squared brackets is the difference in RMSE expressed as a percentage of the signal’s range). The predictions of ankle kinetics further improved when lowering the Achilles tendon stiffness (RMSE decreased from 8.2 to 6.3 Nm [-6.1%]). We believe we did not emphasize enough in the original submission of this paper that although ankle kinematics got slightly worse, ankle kinetics got better. We have made that clearer in the revised version of the manuscript.

Overall, we do not believe that reproducing knee kinematics is more important than reproducing ankle kinematics. But here, adding toes improved knee kinematics and kinetics as well as ankle kinetics, at the slight expense of ankle kinematics. We therefore believe that adding toes to the model is important in addition to being physiological relevant (humans have toes). Furthermore, from a qualitative point of view, stance ankle kinematics was already pretty bad in the first place (i.e., with the model without toes) and adding toes only made it slightly worse. We hypothesize, as described in the paper, that the poor ankle kinematics might be caused by other modeling limitations.

In the revised version of the paper, we clearly acknowledge that ankle kinematics got worse when adding toes to the model, but also further emphasize how ankle kinetics improved.

Changes in abstract (lines: 28-32)

*Modeling toes also slightly decreased the first vertical ground reaction force peak, increasing its agreement with experimental data, and improved stance ankle kinetics. It nevertheless slightly worsened predictions of ankle kinematics.* *Decreasing Achilles tendon stiffness improved the realism of ankle kinematics, but there remain large discrepancies with experimental data.*

Changes in results (lines: 282-289)

*Predictive simulations with a model incorporating toe joints produced stance knee flexion angles, knee extension torques, and ankle flexion torques that better reproduced experimental data as compared to simulations produced with a model with locked toe joints (RMSE decreased from 6.8 to 5.3° [-4.5%], from 17.8 to 10.6 Nm [-11.2%], and from 17.0 to 6.2 Nm [-11.2%] for the stance knee kinematics, knee kinetics, and ankle kinetics, respectively) (Figs 1-2; results for all joints and muscles are shown in S1-3 Figs; animations are shown in S1-2 Movies). The predicted stance ankle kinematics changed when incorporating toe joints but slightly worsened compared to experimental data (RMSE increased from 7.5 to 8.2° [2.4%]).*

Changes in discussion (lines: 342-348)

*our study reveals that a more physiologically plausible model of the feet, which incorporate toe joints, contributes to robustly eliciting knee flexion during stance but also improves stance ankle kinetics and reduces overestimation of the first vertical ground reaction force peak. Adding toe joints slightly worsened predictions of ankle kinematics, which were already of limited accuracy when using a model without toe joints. Decreasing Achilles tendon stiffness by up to 60% slightly improved the realism of the simulated ankle kinematics, but further reduction drastically altered the simulated gait pattern.*

Changes in conclusion (lines: 463-469)

*Incorporating toes in the musculoskeletal model contributed to robustly eliciting stance knee flexion, improved ankle and knee kinetics, and normalized the first vertical ground reaction force peak. Yet it did not improve stance ankle kinematics. In contrast, decreasing the stiffness of the Achilles tendon by up to 60% slightly improved stance ankle kinematics, but there remain large discrepancies with experimental data. The poor predictions of ankle kinematics in simulations of walking thereby remain an open question, and further work is required to better understand how mechanical factors shape human walking.*

Authors compared their simulation with experimental data of one subject.

The author is right that we included experimental data of only one subject. We also scaled the musculoskeletal model to the anthropometry of that particular subject. We therefore expect that if we perfectly captured the neuro-mechanics of gait in our model, our simulation would be closer to the measured experimental data of this subject than to average experimental data from healthy adults. Adding experimental data from other subjects would likely make the experimental standard deviation wider and our simulations look closer to measured data. Only including data from one subject therefore reduces variability and makes discrepancies between simulated and reference data clearer. We believe that future work should generate simulations using models from different subjects to evaluate how simulations capture inter-subject variations, but such analysis was out of the scope of the present study. We acknowledged that we used data from only one subject and discussed the aforementioned avenue for future work in the revised discussion.

Changes in discussion (lines: 394-397)

*Furthermore, we used data and models scaled based on anthropometric data from only one subject. Future studies could include more subjects and evaluate whether simulations capture inter-subject variability, which could help elicit the origins of variability (e.g., differences in motor control objectives and mechanics).*

There is no information of number of recorded experimental cycles.

Experimental data were averaged over 10 gait cycles. We have added this information in the methods.

Changes in methods (lines: 149-150)

*We used experimental data from a single subject averaged over 10 gait cycles for comparison with simulation outcomes*

On figures, experimental data represented only by shaded area without mean pattern.

We have adjusted all figures such that the mean pattern is represented.

On some panels it is impossible to see how experimental patterns look like. For example, subtalar angle on S2Fig. There is no information where stance and swing phases on figures.

We agree with the reviewer that experimental patterns were sometimes hard to see. In this revised version, we added mean patterns, as suggested by the reviewer, as well as vertical lines to distinguish stance and swing (to facilitate the reading, we only plotted the stance to swing transition derived from the experimental data). We believe those changes will make the experimental patterns easier to see.

Regarding the particular case of subtalar torques (S2 Fig), the experimental torques (i.e., from inverse dynamics) are very noisy, explaining why the mean +/- two standard deviations depicted in the figure covers such a wide area. We have double checked our data and found nothing wrong in our methodology. We believe the large variation in subtalar torques is due to high sensitivity of the subtalar torque to COP position and subtalar angle. The subtalar angle is known to be hard to capture with optical motion capture, which might give rise to inaccuracies in subtalar torques. Further, since the joint axis is very close to the COP, slight inaccuracies in COP position with respect to the location of subtalar axis might have a large effect on joint torque as well. To avoid any confusion, we have removed the experimental data for the subtalar torques and motivated this choice in the caption.

Changes in S2 Fig caption (lines: 494-496)

*The experimental data for subtalar torques are not shown given the large variability, which we suspect is due to measurement and modeling errors in the inverse dynamic analysis rather than representing true variability.*

**Bibliography**

1.	Lai A, Lichtwark GA, Schache AG, Lin YC, Brown NAT, Pandy MG. In vivo behavior of the human soleus muscle with increasing walking and running speeds. J Appl Physiol. 2015;118(10):1266–75.

2.	Roberts TJ. Contribution of elastic tissues to the mechanics and energetics of muscle function during movement. J Exp Biol. 2016;219(2):266–75.

3.	Fukunaga T, Kubo K, Kawakami Y, Fukashiro S, Kanehisa H, Maganaris CN. In vivo behaviour of human muscle tendon during walking. Proc R Soc B Biol Sci. 2001;268(1464):229–33.

4.	Ishikawa M, Pakaslahti J, Komi P V. Medial gastrocnemius muscle behavior during human running and walking. Gait Posture. 2007;25(3):380–4.

5.	Zajac FE. Muscle and tendon: properties, models, scaling, and application to biomechanics and motor control. Crit Rev Biomed Eng. 1989;17(4):359–411.

6.	Delabastita T, Afschrift M, Vanwanseele B, De Groote F. Ultrasound-based optimal parameter estimation improves assessment of calf muscle–tendon interaction during walking. Ann Biomed Eng. 2020;48(2):722–33.

7.	Swinnen W, Hoogkamer W, De Groote F, Vanwanseele B. Habitual foot strike pattern does not affect simulated triceps surae muscle metabolic energy consumption during running. J Exp Biol. 2019;22(23):1–8.

8.	Lichtwark GA, Wilson AM. Interactions between the human gastrocnemius muscle and the Achilles tendon during incline, level and decline locomotion. J Exp Biol. 2006;209(21):4379–88.

9.	Dembia CL, Bianco NA, Falisse A, Hicks JL, Delp SL. OpenSim Moco: Musculoskeletal optimal control. PLoS Comput Biol [Internet]. 2020;16(12 December):1–21.

10.	Delp SL, Loan P, Hoy MG, Zajac FE, Topp EL, Rosen JM. An interactive graphics-based model of the lower extremity to study orthopaedic surgical procedures. IEEE Trans Biomed Eng. 1990;37(8):757–67.

11.	Inman VT. The joints of the ankle. Baltimore: Williams & Wilkins; 1976.

12.	Sasaki K, Neptune RR, Kautz SA. The relationships between muscle, external, internal and joint mechanical work during normal walking. J Exp Biol. 2009;212(5):738–44.

13.	Lloyd DG, Besier TF. An EMG-driven musculoskeletal model to estimate muscle forces and knee joint moments in vivo. J Biomech. 2003;36(6):765–76.

14.	Sartori M, Reggiani M, Farina D, Lloyd DG. EMG-driven forward-dynamic estimation of muscle force and joint moment about multiple degrees of freedom in the human lower extremity. PLoS One. 2012;7(12):1–11.

15.	Bhargava LJ, Pandy MG, Anderson FC. A phenomenological model for estimating metabolic energy consumption in muscle contraction. J Biomech. 2004;37(1):81–8.

16.	Falisse A, Serrancolí G, Dembia CL, Gillis J, Jonkers I, De Groote F. Rapid predictive simulations with complex musculoskeletal models suggest that diverse healthy and pathological human gaits can emerge from similar control strategies. J R Soc Interface. 2019;16(157):20190402.

17.	Hamner SR, Seth A, Delp SL. Muscle contributions to propulsion and support during running. J Biomech. 2010;43(14):2709–16.

18.	Hogan N. Adaptive control of mechanical impedance by coactivation of antagonist muscles. IEEE Trans Automat Contr. 1984;29(8):681–90.

19.	Stroeve S. Impedance characteristics of a neuromusculoskeletal model of the human arm I. Posture control. Biol Cybern. 1999;81(5–6):475–94.
