# Supplementary material for: Modeling toes contributes to realistic stance knee mechanics in three-dimensional predictive simulations of walking
Source: PLoS One. 2022 Jan 25;17(1):e0256311. doi: 10.1371/journal.pone.0256311 (PMC8789163; doi:10.1371/journal.pone.0256311)
Supplement: Attachment — Submitted filename: Response To Reviewers.docx [file pone.0256311.s011.docx]

Dear Editor,

Many thanks for giving us a chance to resubmit this manuscript. We have addressed the remaining comments. We hope the paper now satisfies the standards of the journal, but we would be happy to clarify certain parts if deemed necessary. You will find our answers to the reviewers’ questions in blue below, with modified text in red.

Yours sincerely,

Antoine Falisse on behalf of all co-authors.

Reviewer #1: The authors have addressed all the comments and critiques, greatly improving the manuscript. There are a couple minor points that be addressed.

We would like to thank the reviewer for all the feedback they provided. We have addressed the remaining points, as discussed below.

“We specified the number of muscles in the revised version of the methods. We think listing all 24 muscles densify the text a lot while not adding much to the story. We therefore only indicated that those were 24 muscles in the lower limbs. Please note that electromyography was only used to evaluate the simulated muscle activations as now explicitly mentioned.” – The muscles are important to list in the methods because they identify the modelled muscles, which have been evaluated in the manuscript and which have been not. This may provide the directions for the future analysis of the model, e.g., which modelled muscle activity has not been validated.

We agree with the reviewer. We have listed all muscles with available EMG in the revised methods.

Changes in revised methods (lines 155-159):

*…from 24 lower limb muscles (hamstrings lateralis and medialis [left and right], gluteus medius [left and right], adductor longus [left], tensor fasciae latae [left], vastus lateralis and medialis [left and right], rectus femoris [left and right], tibialis anterior [left and right], gastrocnemius lateralis [left and right] and medialis [left], soleus [left and right], and peroneus longus [left and right] and brevis [left]).*

“The formula for the metabolic energy rate is described in length in the cited publication [15], and we believe it is out of the scope of this paper to provide it again here, as it involves many terms with many variables that would need to be defined. Yet we revised this part of the methods to make clearer what terms were included in the computation of the metabolic energy rate.” – The comment from the first review was more to do with the approximation than the formula itself. Since not the original formula from Bhargava et al., 2004 is used, but an approximation of it, which is not described elsewhere, it should be provided in the manuscript for reproducibility, or the way it differs from the original.

Sorry that we misunderstood the original comment. We agree that it makes sense to provide more details about the smooth approximation of the original model. We have added a detailed description of the formula for the metabolic energy rate in S1 Appendix, and we refer to it in the manuscript.

Changes in revised methods (line 182):

*…(details about model implementation in S1 Appendix).*

Changes in revised supporting information (lines 486-487):

*S1 Appendix: Effect of the toe joint damping value on the predicted walking pattern and details about the smooth approximation of the metabolic energy model.*

Reviewer #2: All comments have been addressed. Authors added all necessary comments and explanations as well as modified figures as requested

We would like to thank the reviewer for all the feedback they provided.
